# Review of the endocrine organ–like tumor hypothesis of cancer cachexia in pancreatic ductal adenocarcinoma

**DOI:** 10.3389/fonc.2022.1057930

**Published:** 2022-11-17

**Authors:** Ying-Chun Yu, Azaj Ahmed, Hsueh-Chou Lai, Wei-Chung Cheng, Juan-Chern Yang, Wei-Chun Chang, Lu-Min Chen, Yan-Shen Shan, Wen-Lung Ma

**Affiliations:** ^1^ Department of Medical Research, Department of Obstetrics and Gynecology, Department of Gastroenterology, and Chinese Medicine Research and Development Center, China Medical University Hospital, Taichung, Taiwan; ^2^ Graduate Institute of Biomedical Sciences, Center for Tumor Biology, School of Medicine, China Medical University, Taichung, Taiwan; ^3^ School of Chinese Medicine, China Medical University, Taichung, Taiwan; ^4^ Division of General Surgery, Department of Surgery, National Cheng Kung University Hospital, College of Medicine, National Cheng Kung University, Tainan, Taiwan; ^5^ Institute of Clinical Medicine, College of Medicine, National Chen Kung University, Tainan, Taiwan; ^6^ Department of Nursing, Asia University, Taichung, Taiwan

**Keywords:** pancreatic ductal adenocarcinoma (PDAC), cachexia, muscle wasting, tissue wasting, endocrine organ-like tumour (EOLT)

## Abstract

Pancreatic ductal adenocarcinoma (PDAC) is one of the most fatal types of solid tumors, associated with a high prevalence of cachexia (~80%). PDAC-derived cachexia (PDAC-CC) is a systemic disease involving the complex interplay between the tumor and multiple organs. The endocrine organ–like tumor (EOLT) hypothesis may explain the systemic crosstalk underlying the deleterious homeostatic shifts that occur in PDAC-CC. Several studies have reported a markedly heterogeneous collection of cachectic mediators, signaling mechanisms, and metabolic pathways, including exocrine pancreatic insufficiency, hormonal disturbance, pro-inflammatory cytokine storm, digestive and tumor-derived factors, and PDAC progression. The complexities of PDAC-CC necessitate a careful review of recent literature summarizing cachectic mediators, corresponding metabolic functions, and the collateral impacts on wasting organs. The EOLT hypothesis suggests that metabolites, genetic instability, and epigenetic changes (microRNAs) are involved in cachexia development. Both tumors and host tissues can secrete multiple cachectic factors (beyond only inflammatory mediators). Some regulatory molecules, metabolites, and microRNAs are tissue-specific, resulting in insufficient energy production to support tumor/cachexia development. Due to these complexities, changes in a single factor can trigger bi-directional feedback circuits that exacerbate PDAC and result in the development of irreversible cachexia. We provide an integrated review based on 267 papers and 20 clinical trials from PubMed and ClinicalTrials.gov database proposed under the EOLT hypothesis that may provide a fundamental understanding of cachexia development and response to current treatments.

## 1 Introduction

For Pancreatic ductal adenocarcinoma (PDAC) is currently the fourth most common cause of cancer-related deaths worldwide and is projected to become the second most common cause of cancer-related deaths by 2030 (1). Due to its aggressiveness and poor prognosis, mortality remains alarmingly high among patients diagnosed with PDAC. Approximately 80%–85% of PDAC patients are diagnosed at advanced stages with unresectable or metastatic tumors, resulting in a 5-year survival rate below 10% (2). During early-stage PDAC, surgical resection is currently the only curative option, although chemotherapy and radiation therapy are also used as primary treatment options, with or without surgery. However, single-agent chemotherapies are rarely effective in PDAC (3). In general, chemotherapy regimens are not universally effective in PDAC and are associated with significant adverse effects, including the development of PDAC-derived cachexia (PDAC-CC), and cachexia occurs in 32%~71% of patients within 12 to 48 weeks of chemotherapy initiation (4).

Patients with PDAC experience a high prevalence (up to 80%) of cachexia, often with early onset (45% of PDAC patients present with cachexia at the time of diagnosis (5)), which may account for up to 30% of mortality (6). Cachexia is defined as the progressive loss of muscle mass and function (6, 7) and is a catabolic multi-organ syndrome characterized by non-volitional weight loss (muscle or adipocyte loss), adipopenia, fatigue, weakness, loss of appetite, and early satiety (8, 9). When muscle mass loss, it enhances chemo-toxicities and insensitivities, contributing to poor overall survival (10).

In general, tumors demand a high energy supply and can promote the wasting of peripheral tissues *via* hyper-catabolism. Tumors compete with other organs/tissues for energy and nutrients, resulting in elevated resting energy expenditure and inducing a negative energy balance. Energy utilization in tumors also results in increased proteolysis and lipolysis combined with decreased lipogenesis and protein synthesis (8, 9, 11, 12). These metabolic reprogramming effects, combined with poor appetite, lead to rapid weight loss among PDAC patients and can contribute to deterioration in the overall quality of life (QoL) and overall survival (OS) (7, 13–15). The complex, multifactorial nature of the metabolic disruptions in cachexia makes effective treatment challenging. The current lack of consensus regarding how to define cachexia and a scarcity of strong evidence produced by robust, rigorous, and mechanistic studies have limited the development of effective treatments (16). In addition, most cachexia studies focus on symptoms associated with individual organs (such as tumor, muscle, or adipocyte tissues) without considering consider systemic interactions. In this review, we provide an up-to-date overview of current cachexia research in PDAC to provide insight regarding the cachexia mediators that act in different organs and explore whether the endocrine organ–like tumor (EOLT) hypothesis of PDAC can explain the development of systemic complications.

## 2 Cachexia criteria and stages

Cancer-derived cachexia (CC) is a multifactorial syndrome involving various metabolic changes in several tissues and organs (8, 9, 12, 17–19). Although patients with pancreatic cancer show a wide range of nutritional alterations, the primary symptom is progressive weight loss due to the loss of skeletal muscle mass, with or without the accompanying depletion of adipose tissue (6, 19–23). Other PDAC-CC-related clinical manifestations include inflammation (24–26), anorexia (27, 28) and metabolic reprogramming (9, 29, 30) etc.,

Numerous studies also focus on exploring new PDAC-CC cachectic mediators, corresponding metabolic functions, and the collateral impacts on wasting organs. A systematic review also suggested a network of cytokines (interleukin [IL]-6, tumor necrosis factor-alpha [TNFα], and IL-8) that may be associated with cachexia development (31). Sah et al. (19) suggested that PDAC-CC can be categorized by three distinct metabolic phases: Phase 1 represents the earliest metabolic change, characterized by new-onset hyperglycemia; Phase 2 is associated with a greater than 5% reduction in body weight with pre-cachectic symptoms (appetite loss and impaired glucose metabolism), suggesting the initiation of cachexia; and Phase 3 is associated with dramatic reductions in all monitored metabolites, lipids, subcutaneous fat, and muscle, except fasting glucose.

Traditionally, a Body Mass Index (BMI) < 18.5 kg/m^2^ was accepted as a marker of being cachectic. However, sarcopenic obesity can be observed in CC, suggesting that weight loss might not be a defining factor (32). According to the most common consensus, published by Fearon et al. (33), the current standard diagnostic criterion for cachexia is represented by percentage of weight loss, BMI values and metabolic changes (29, 33, 34). Simply, CC were classified into three stages: pre-cachexia, cachexia, and refractory cachexia (**Table 1**). This classification currently did not fully applicable in clinics but is rather to be considered as a proposal under evaluation. Additional parameters (**Table 2**) have been developed to improve diagnosis, such as food intake measures, albumin levels, anorexia assessment, markers of systemic inflammation (CRP >10 mg/L), muscle mass measurements, the Skeletal Muscle Index (SMI), bioelectrical impedance analysis (BIA), the Fat-Free Mass Index and cachexia index (CXI). Although these diagnostic measurements did not include in the latest consensus, they suggested that several effective parameters could more accurately identify cachexia. A recent systematic review by Paval et al. described the between-study inconsistencies in grouping criteria as a major hindrance to the conduct of meta-analyses for cachexia (31). Refined CC-criteria is critical for evaluating the response to cachexia/antitumor therapy. Because early-onset PDAC-CC can present concomitant with the detection of the primary tumor burden, but cachexia can continue even after the tumors have been surgically removed or effectively treated (15). Patients received either preoperative surgery or chemotherapy/chemoradiation; unintended weight loss coupled with muscle wasting can often be observed, contributing to poor outcomes in PDAC (10, 13, 15, 29). There is no effective strategy to mitigate refractory PDAC-CC. Therefore, the early and precise identification of PDAC-CC is needed to estimate prognosis and prevent progression to the refractory cachexia. More practical, longitudinal definitions of cachexia remain necessary that consider all aspects of the cachexia phenotype.

**Table 1:** Cachexia criteria/definition.

Score system	Criteria	Ref
BMI	BMI <18.5 kg/m^2^	(35)
Body Weight	-Weight loss ≥10%;	(36)
	-Presence of at least 1 symptom: anorexia, fatigue, or early satiation	
EPCRC	• **Pre-cachexia**: Weight loss ≤5%, anorexia, metabolic changes	(37, 38)
	• **Cachexia:** Weight loss >5% over past 6 months , or BMI < 20kg/m^2^ and weight loss >2% , or sarcopenia and weight loss >2% (Skeletal muscle index: males <7.26 kg/m^2^; females <5.45 kg/m^2^)	
	• **Refractory Cachexia:** Variable degree of cachexia cause poor survival and not responsive to anticancer treatment.	
Glasgow Prognostic Score	CRP >10 mg/L	(39)
Cancer Cachexia Study Group (CCSG)	Multifactorial syndrome: Weight loss, reduce food intake, systematic inflammation CRP >10 mg/L, weight loss >10%, energy intake <1500 kcal/day	(37)
Cachexia Score (CASCO)	Body weight and lean body mass loss; anorexia; inflammatory, immunological and metabolic disturbances; physical performance and QoL.	(40)
Cachexia definition	A complex metabolic syndrome associated with underlying illness and characterized by loss of muscle with or without loss of fat mass, including weight loss (>5%), decreased muscle strength, reduced muscle mass, anorexia, symptoms of fatigue, or biochemical abnormalities (anemia, inflammation CRP >5mg/L, or low albumin).	(41)
Cachexia staging score (CSS)	Defined by 5 components: Weight loss in 6 months, appetite loss, SARC-F questionnaire assessing muscle function and sarcopenia, ECOG performance status, abnormal biochemistry	(42)
Cachexia index (CXI)	Defined by reduced muscle mass (SMI: skeletal muscle index), poor nutritional status (Alb: serum albumin g/dL), and systemic inflammation (NLR: neutrophil-to-lymphocyte ratio). $\text{CXI}=\frac{\text{SMI}\times \text{Alb}}{\text{NLR}}$	(43, 44)

BMI, body mass index; CRP, C-reactive protein; EPCRC, European Palliative Care Research collaborative; CASCO, Cachexia Score.

**Table 2:** Cachexia assessment.

Assessment	Method	Ref
Food intake	PG-SGA-SF: Patient-Generated Subjective Global Assessment Short-Form Ingesta score MNA-SF: Mini Nutritional Assessment Short-Form NIS: Nutritional impact symptoms EORTC QLQ-CAX24 Questionnaire	(35, 45–48)
Anorexia	FAACT: Functional assessment of anorexia/cachexia treatment	(49)
Inflammation	modified Glasgow prognostic score	(50)
Body Weight	-Weight loss ≥10% -Presence of at least 1 symptom: anorexia, fatigue, or early satiation Weight Loss Grading System (WLGS 0, 1, 2, 3, or 4)	(36, 51)
Muscle mass	Muscle mass: mid-upper arm muscle area (men <32 cm^2^; women <18 cm^2^)	(52, 53)
Skeletal Muscle Index	Computed tomography (men <36.54–45.40 cm^2^/m^2^; women <30.21–36.05 cm^2^/m^2^)	(54)
Body composition (body fat and muscle mass)	Dual-energy X-ray absorptiometry (men <5.86–7.40 kg/m^2^; women <4.42–5.67 kg/m^2^) Bioelectrical impedance analysis (men <6.75–7.40 kg/m^2^; women <5.07–5.80 kg/m^2^) Fat-Free Mass Index	(53, 55, 56)
Fatigue	Single Item Fatigue (SIF)	(57)
Malnutrition assessment	PINI: Prognostic Inflammation Nutrition Index CRP (mg/L) × α1-acid glycoprotein]/[albumin (g/L) × transthyretin (g/L)] NRI: Nutritional Risk Index NRI = 1.519× albumin (g/L) + 0.417× (current weight/usual weight ×100). Criteria: >100: no malnourishment 97.5-100: mild malnourishment 83.5-97.4: modern malnourishment < 83.5: severe malnourishment. low albumin (<35 g/L); CRP (>5 or >10 mg/L) transthyretin (prealbumin): low transthyretin (variously <110 or <180mg/L)	(58, 59)
Energy expenditure	Harris Benedict formula: Men: BMR = 66.5 + (13.76 × weight in kg) + (5.003 × height in cm) – (6.755 × age) Women: BMR = 655.1 + (9.563 × weight in kg) + (1.850 × height in cm) – (4.676 × age)	(60)

## 3 EOLT hypothesis in PDAC-CC

The EOLT hypothesis was proposed to explain how tumor tissues drive disease progression, including CC (31). The EOLT hypothesis states that the tumor acts as an endocrine organ, resulting in dynamic bi-directional communications between the tumor microenvironment (TME) and various organs, leading to the regulation of macroenvironmental changes.

**Figure 1:**
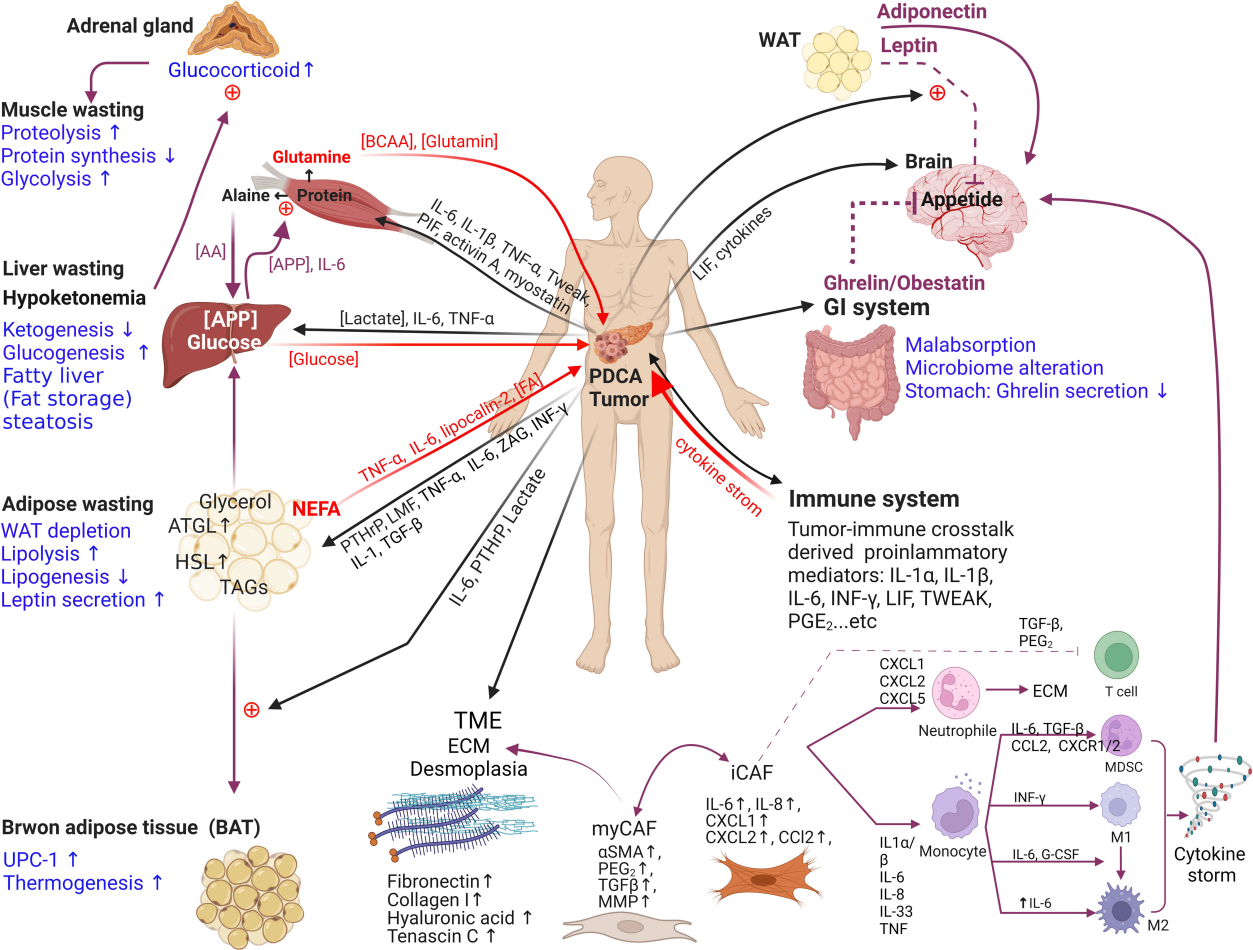
The endocrine organ–like tumor (EOLT) hypothesis for multifactorial cachexia syndrome. EOLT states that solid tumors create multiple endocrine/paracrine organs which differs from the ‘‘seed and soil’’ hypothesis. Tumor-organ crosstalk and interorgan signal crosstalk did not rely on reshaped tumors only. Mostly influenced by different cachectic regulators, such as tumor-derived factors, pro-inflammatory immune mediators (ie. IL-6, IL-1α, IL-1β, TNFα, IFN-γ, ZAG, PIF, activin A, LIF, TWEAK, PGE_2_), and hormones (including glucocorticoids and PTHrP). These cachectic mediators act as paracrine/autocrine manners, trigger positive feedback to other organs and form a bidirectional circuit (black arrow means mediators derived from tumor; red arrow means mediators derived from peripheral tissues/organs; purple arrow means influence between peripheral tissues/organs). When the communication between tumor and organs exists, metabolic reprogramming (mark in blue: glycolysis↑, proteolysis↑, lipolysis↑ and gluconeogenesis↑) produces bidirectional positive feedback to other organs in cachexia. For example, inflammatory cytokines increase lipolysis in white adipose tissue (WAT), releasing free fatty acids (FAs) that further fuel tumor growth and promote muscle wasting (18–21, 61, 62). Adipocyte also can secrete adipokines (e.g., leptin, adiponectin, and lipocalin-2), IL-6, and TNFα which release *via* extracellular vesicles (EVs) into the circulation to influence the TME or mediate the appetite (61, 63, 64). Similarly, muscle wasting regulates by hormones, adipocyte-derived mediators and tumor-derived factors (65). Cachexia is a wasting disease that represents metabolic disruptions driven by systemic inflammation and is characterized by the depletion of adipose tissue and skeletal muscle Interleukin, IL; tumor necrosis factor-alpha, TNFα; interferon-gamma, IFN-γ; zinc alpha 2-glycoprotein, ZAG; proteolysis-inducing factor, PIF; leukemia inhibitory factor, LIF; TNF-related weak inducer of apoptosis, TWEAK; prostaglandin E_2_, PGE_2_; tumor-derived parathyroid hormone–related protein, PTHrP; amino acid, AA; acute phase protein, APP; triglycerides, TAG; brown adipose tissue, BAT; white adipose tissue, WAT; uncoupling protein 1, UCP1; extracellular matrix, ECM; branched-chain amino acids, BCAAs; chemokine (C-X-C motif) ligand, CXCL; matrix metalloproteinases, MMPs; α-smooth muscle, α-SMA; tumor microenvironment, TME.

PDAC-CC results in systemic wasting and involves multiple organ dysfunction (**Figure 1**), accompanied by symptoms including poor appetite, fatigue, depression, muscle wasting, fat wasting, malabsorption, and constipation (**Table 3**). Tumors secrete cachexia-inducing factors and stimulate host–tumor interactions involve cancer-organ metabolic reprogramming and interorgan signal crosstalk in tumor progression and cachexia development (21, 32). For example, tumor-derived cytokines induce systemic inflammation, stimulating the release of neuropeptides that lead to poor appetite, and the resulting anorexia exacerbates tissue wasting (6, 14, 26). With cachectic environment, adipose and muscle tissues can act like paracrine/endocrine organs in response to cachectic factors, providing reciprocal regulation of energy expenditure and cachexia process (8, 9, 17, 33). Cachexia is a wasting disease that represents metabolic disruptions, mainly catabolisms, driven by systemic inflammation and is characterized by skeletal-muscle proteolysis, adipose tissue lipolysis and hepatic gluconeogenesis (**Figure 1**) (20, 22, 24, 34). These inter-organ interactions affect metabolisms in the formation of feedback loops. Thus, PDAC-CC can be characterized by two interacting dimensions:

**1.** Systemic metabolic changes, often associated with *KRAS* mutations (genetic instability).
**2.** Pro-cachectic mediators and microRNAs (miRNAs) exacerbated in metabolic disruptions.

**Table 3:** The multi-organ response in PDAC-derived cachexia.

Organ	Tissue alterations	Main implications
Brain	• Alterations in appetite • Alterations in taste and smell	• Anorexia • Negative energy balance
Gut	• Changes in microbiota • Altered ghrelin production • Gut barrier dysfunction	• Malabsorption
Liver	• Production of acute phase proteins • Decreased albumin production • Increase gluconeogenesis (increase Cori cycle)	• Acute phase response •Negative energy balance
Skeletal muscle	• Increased proteolysis • Increased glycolysis • Decreased protein synthesis	• Wasting • Atrophy, sarcopenia • Fatigue • Decreased physical performance
White adipose tissue (WAT)	• Activation of thermogenesis • Increased lipolysis • Increased leptin secretion • Release of fatty acids • ‘Browning’	• WAT depletion • Decreased food intake and body weight
Brown adipose tissue (BAT)	• Activation of thermogenesis	• Energy expenditure
Pancreatic insufficiency	• Endocrine dysfunction • Pancreatic exocrine insufficiency (PEI)	• Low insulin production • Malabsorption

PDAC, pancreatic ductal adenocarcinoma.

Citation reference (6, 8, 9, 14, 25–27, 66–81).

The high prevalence of cachexia in PDAC is associated with distinct metabolic effects mediated by tumor created environments, including *KRAS* mutations, pro-cachexia mediators, and alteration in pancreas and liver. The present review summarizes the current understanding of PDAC-CC according to the EOLT hypothesis.

### 3.1 Metabolic alterations and high energy demands in tumors

**Figure 2:**
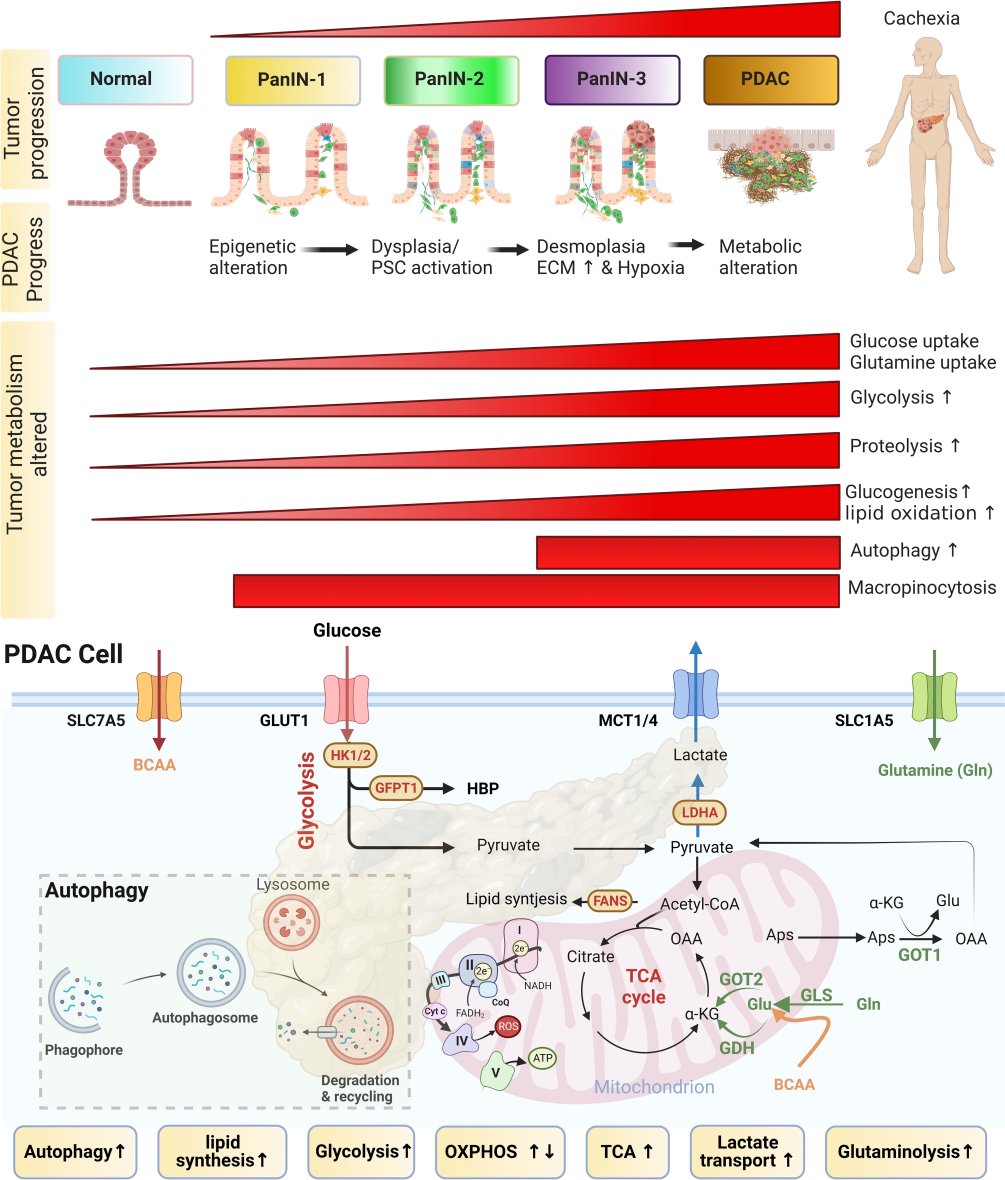
Metabolism alterations in PDAC-CC. The upper panel shows that pancreatic ductal adenocarcinoma (PDAC)-derived cachexia (PDAC-CC) arises from the multi-stage progression of precursor lesions, known as pancreatic intraepithelial neoplasia (PanIN). PanINs are characterized by a continuum of increasingly stroma features (from low-grade dysplasia developing to high-grade desmoplasia). A desmoplastic response induced a fibro-inflammatory microenvironment, stimulating an aberrant metabolic response that is associated with cachexia. During early-stage PDAC, histology can be used to identify several distinct types of precursor lesions. The most common types are microscopic PanIN, low-grade (PanIN-1 and PanIN-2), and high-grade (PanIN-3). The progression to PanIN and to PDAC is associated with cachexia development. Multiple metabolic alterations follow the progression of cachexia, resulting in the reprogramming of glucose, amino acid, and lipid pathways. Metabolic alterations include nutrient scavenging pathway), such as glycolysis glutaminolysis, autophagy, proteolysis, lipid oxidation, and micropinocytosis (Most of them were upregulated during the development of cachexia). However, early-onset cachexia can arise independent of the PDAC stage, occurring in the pre-diagnostic PDAC stage. More than one-third of cancer patients were malnourished before chemotherapy, implying that the cachexia occurred early and followed a poor response to chemotherapy. Interestingly, some of cachexia occurs after the chemotherapy. **The lower panel:** The metabolic alterations, including increase glycolysis, glutaminolysis, lactate transport and autophagy … etc, in PDAC cell associated with PDAC-CC, primarily due to promote the expression in key enzymes (HK1/2, GFPT1, and LDHA) and transporters (GLUT1, MCT1/4, SLC7A5, and SLC1A5). The metabolic shift from the tricarboxylic acid (TCA) cycle and oxidative phosphorylation (OXPHOS) to aerobic glycolysis is tightly regulated. HK1/2, hexokinase; GFPT1, glutamine fructose 6-phosphate amidotransferase 1; LDHA, lactate dehydrogenase A; GLUT1, Glucose transporter 1; MCT1/4, monocarboxylate transporter 1/4; SLC7A5 (LAT1), neutral amino acid antiporter; SLC1A5, glutamine transporter.

PDACs are characterized by high energy demands within a nutrient-deprived microenvironment. Aggressive PDAC is characterized by increased glycolysis and glutamine metabolism, closely associated with downstream anabolic pathways in the tumor’s hypoxic desmoplastic environment (8, 9, 27, 29, 63). The deprivation of glucose and glutamine and lactic acidosis promote glycolytic and glutaminolysis activity (61, 82, 83). Metabolic alterations are hallmarks of PDAC and PDAC-CC, particularly the dysregulation of glucose and glutamine metabolism (8, 9, 14, 19, 29, 62). However, PDACs under different oxygen and nutrition conditions show distinct and heterogeneous metabolite profiles associated with aerobic glycolysis (the Warburg effect), OXPHOS (oxidative phosphorylation; also known as the reverse Warburg effect), lipid dependence, autophagy, and glutaminolysis (**Figure 2**). Metabolic alterations are positively correlated with high-grade pancreatic intraepithelial neoplasia (PanIN-3). However, early-onset cachexia also develops independent of PDAC, occasionally presenting in the pre-diagnostic PDAC stage (4). Cachexia is a metabolic disorder involving several nutrient scavenging pathways, including autophagy, micropinocytosis, glycolysis, lipid oxidation, and micropinocytosis (**Figure 2** **: upper panel**).

**Figure 3:**
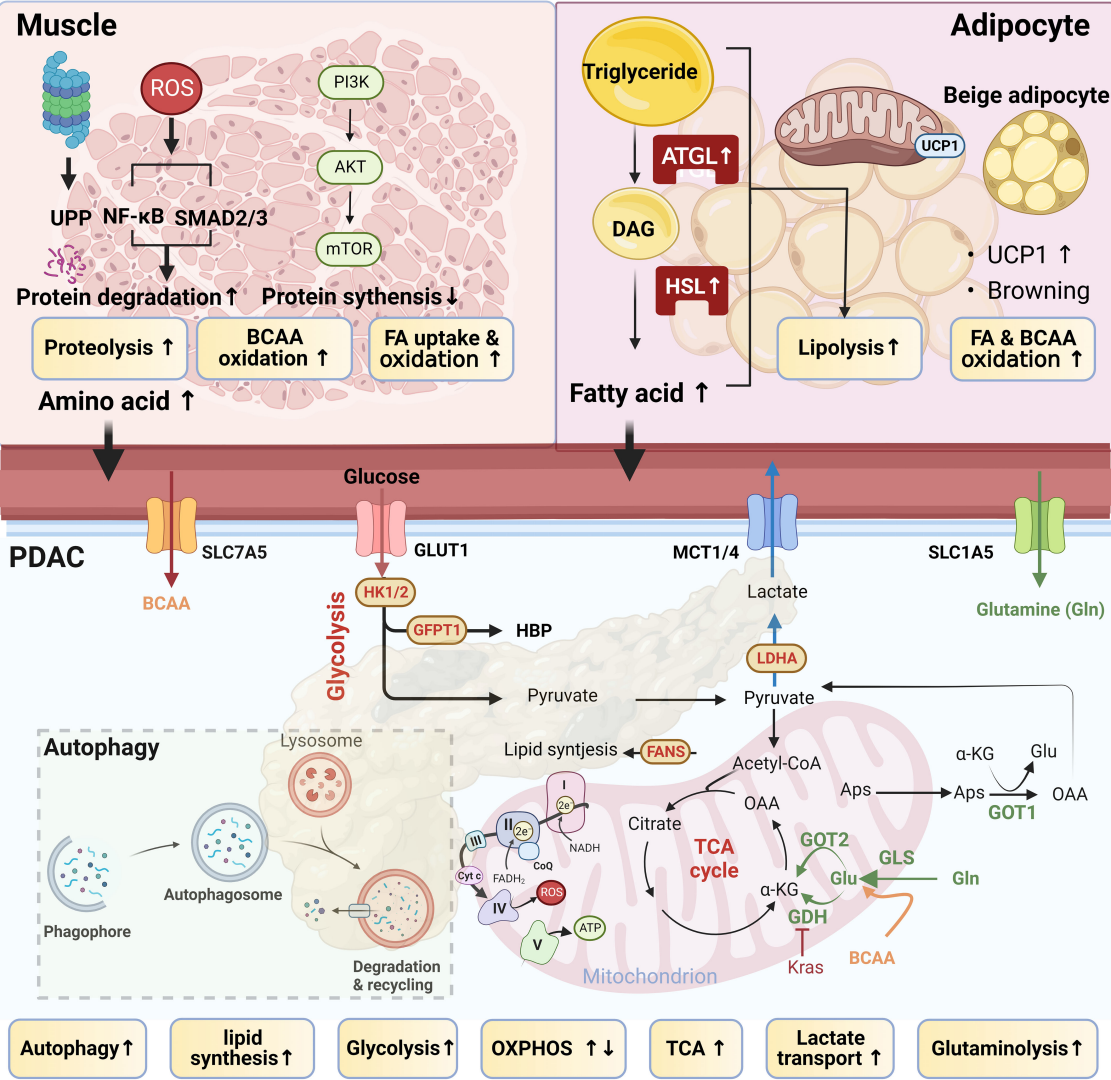
Tumor–muscle–adipocyte crosstalk. Pancreatic ductal adenocarcinoma (PDAC)-derived cachexia occurs due to a feedback circuit among tumor, adipocyte, and muscle tissues. In PDAC, tumor‐derived factors, including interleukin (IL)‐1, IL-6, IL-8, proteolysis-inducing factor (PIF), lipid mobilization factor (LMF), and tumor necrosis factor‐α (TNFα), enhance proteolysis, lipolysis, and the catabolic state of muscle and adipocytes, leading to adipose and muscle wasting. Tumor cell–triggered metabolic reprogramming in muscle and adipocytes releases metabolic products, such as branched-chain amino acids (BCAA) and free fatty acids (FFAs), to fuel tumor growth. Lipolysis and proteolysis are the two main processes causing adipose and muscle wasting in cachexia.

PDAC survives and thrives in relatively hypoxic and nutrient-poor niches, driven by [1] reprogramming intracellular nutrient metabolism, including glucose, amino acids, and lipids; [2] scavenging and recycling nutrients; and [3] promoting metabolic crosstalk (**Figures 2** **: lower panel** and **Figure 3**) (8, 9, 62). PDAC-CC exacerbates metabolic reprogramming, promoting the deterioration of muscle and adipose tissue (**Figure 3**), further supporting the energy and nutrient needs of the tumor tissue.

#### 3.1.1 Glucose

Glycolytic flux can result in changes in the pentose phosphate pathway (PPP), the hexosamine biosynthesis pathway (HBP), serine biosynthesis, and the tricarboxylic acid (TCA) cycle, promoting CC development (64). Rate-limiting glycolytic enzymes, such as hexokinase 1/2 (HK1/2), phosphofructokinase 1, and lactate dehydrogenase A (LDHA), are upregulated to facilitate the Warburg effect, resulting in glycolytic flux and the production of lactate from glucose in PDAC-CC (63, 64). The upregulation and translocation of glucose transporters (GLUT1, encoded by *SLC21A*) in tumor tissues facilitate glucose uptake for aerobic glycolysis. Increased glycolytic flux in response to host–tumor interactions in cachexia results in the production of high lactate levels, leading to lactic acidosis. To address lactic acidosis, PDACs robustly express monocarboxylate transporters (MCT1 and MCT4, encoded by *SLC16A1* and *SLC16A3*, respectively) to coordinate glucose utilization and lactate mobilization (65, 84). Other glucose metabolism pathways are also altered in PDAC, such as the upregulation of the rate-limiting enzyme of the HBP, glutamine: fructose-6-phosphate amidotransferase-1 (GFPT1) (64). Many other mediators regulate glucose metabolism in pancreatic cancer cells. Under hypoxic conditions, hypoxia-inducible factor-1 (HIF-1) can promote glycolysis and upregulate the expression of HBP-related enzymes, such as GFPT2, an isoform of the HBP rate-limiting enzyme GFPT1 (63).

In a model of PDAC-CC, athymic mice injected with high-glycolytic MiaPaCa2 cells showed evidence of cachexia, such as weight loss, fat depletion, and muscle proteolysis (66), suggesting that glycolysis may be involved in PDAC-CC development. Glycolysis was associated with inefficient inter-organ substrate shuttles, as assessed by the lactate-to-pyruvate utilization ratio, LDH activity, and MCT1 expression, which was correlated with cachexia-related weight loss (63). The upregulation of GLUT1 and MCT1/4 promotes glucose utilization and improves the lactate-to-pyruvate utilization ratio in tumor tissue (63, 65). Paradoxically, OXPHOS, also known as the reverse Warburg effect, occurs in muscle, resulting in an increased lactate-to-pyruvate production ratio, providing a potential lactate supply for tumor use and supporting tumor progression and consequent atrophy (85). Additionally, the tumor secretes interferon-gamma (IFN-γ), which mediates the development of insulin resistance *via* reduced glucose and fatty acid [FA] uptake, leading to enhanced lipolysis in WAT (86). Inefficient inter-organ substrate shuttles are regarded as hallmarks of EOLT (**Figure 3**).

Aerobic glycolysis occurs more commonly in tumor tissues than OXPHOS, which requires a sufficient oxygen supply, although OXPHOS is more efficient for ATP generation (30, 87). Nutrient depletion forces tumors to adapt by inducing nutrient scavenging mechanisms to support cancer progression, which can lead to CC (8, 9, 88). OXPHOS occurs in the mitochondria and is sensitive to stress conditions, as the respiratory complexes in stressed mitochondria produce high levels of reactive oxygen species (ROS) (62). Autophagy is a stress response induced by ROS to remove damaged mitochondria that overproduce ROS, promoting mitochondrial metabolism (89) (**Figure 2** **: lower panel**). In cachectic patients, increased OXPHOS and dysfunctional autophagy are associated with increased muscle wasting (**Figure 3**) (90). Autophagy is an important proteolysis pathway activated during PDAC-CC and muscle wasting (91, 92).

Tumors supported by an adequate blood supply can perform aerobic metabolism and tend to exhibit the reverse Warburg effect. In tumors, aerobic metabolisms may utilize intermediates, such as lactate, to fuel the TCA cycle (**Figure 2** **: lower panel),** decreasing their dependence on glucose. The low uptake of glucose and the enhanced uptake of intermediate metabolites by tumors under aerobic conditions could protect these tumors from competing with hypoxic tumor regions (such as desmoplastic tumors) for glucose. In addition to OXPHOS, tumors able to perform aerobic metabolism can also utilize glutaminolysis as an alternative energy production pathway requiring activated mitochondrial metabolism (93). Glutamine is the most abundant and versatile nonessential amino acid (NEAA), found in both the blood and the cell cytoplasm, and can be used by both the glutamine-dependent pyruvate cycle and the TCA cycle (30, 94). In contrast, hypovascularization and desmoplasia often occur in PDAC; studies also found that HIF-1α (hypoxia-inducible factor-1α) stabilization promotes glycolytic enzymes to shift the metabolism by repressing OXPHOS (95, 96). Supposing that if the function of glycolysis is weakened, OXPHOS and glutamine-based processes will serve as alternative energy generation mechanisms in glucose-limited tumors (87) (**Figure 2** **: lower panel**).

#### 3.1.2 Amino acids

Altered amino acid (AA) metabolism is a frequent feature in CC. Branched-chain amino acids (BCAA: leucine, isoleucine, and valine) act as important carbon sources and are useful for FA biosynthesis. High BCAA levels in plasma are associated with early PDAC and are often derived from increased protein breakdown in muscle and other body tissues (**Figure 3**) (97, 98). The utilization of BCAAs by PDAC can result in plasma BCAA depletion during late-stage PDAC. Similar observations have been reported for glutamate, in which the plasma levels of glutamate and the glutamine/glutamate (Q/E) ratio are significantly reduced in cachectic patients and animal models compared with their healthy counterparts (83, 99). Glutamine metabolism is a primary source of nitrogen and carbon, contributing to macromolecular synthesis and redox balance (83). Glutaminase 1 (GLS1) converts glutamine to glutamate, after which glutamate dehydrogenase (GDH) catalyzes the conversion from glutamate to α-ketoglutarate (α-KG). However, GDH is repressed in PDAC, and cytoplasmic aspartate transaminase (GOT1) is upregulated (83) (**Figure 2** **: lower panel)**. Cachexia is associated with more aggressive forms of PDAC, which may reflect the increased access of tumor cells to nutrients derived from protein breakdown and systemic changes in glucose metabolism (97, 100). Higher circulating BCAA levels may arise from the impaired catabolism of AAs that are commonly found in muscle (**Figure 3**). Muscle wasting is characterized by decreased muscle mass, increased proteolysis, and reduced protein synthesis, changes which are mediated by the proteasome, nuclear factor kappa B (NF-κB), and the mammalian target of rapamycin (mTOR) pathways. The phosphoinositol 3-kinase (PI3K)/AKT/mTOR pathway is a nutrient-sensing mechanism stimulated by decreased glucose availability in the muscle. A higher mTOR activity induced by *KRAS* mutation in PDAC which is positively correlated to higher circulating BCAA levels (12). mTOR activation is responsible for the uptake of BCAA in tumor tissue. Circulating BCAA also can affect subcutaneous adipocyte AA dysmetabolism. Both NF-κB and AKT/mTOR signaling are involved in proteolysis. NF-κB regulates the ATP-dependent ubiquitin–proteasome proteolytic pathway, including muscle-specific E3 ubiquitin ligases (such as muscle atrophy F box protein [MAFbx/atrogin-1] and muscle RING finger–containing protein 1 [MuRF1]), which promote proteolysis and contribute to muscle atrophy (22). Cachexia is the end result of convergent metabolic adaptations induced by tumors to satisfy their metabolic requirements.

#### 3.1.3 Lipids

In addition to glucose and amino acid metabolism, metabolic alterations in cachexia can include lipid metabolism. Approximately 93% of triacylglycerol FAs used by tumors are synthesized *de novo* by the mitochondria and cytosolic acetyl coenzyme A (CoA). Enzymes that participate in *de novo* FA and cholesterol synthesis are upregulated in PDAC, such as FA synthase (FASN) and 3-hydroxy-3-methylglutaryl coenzyme A reductase (HMGCR). Under pancreatic inflammatory conditions, wasting adipocytes release FAs into the plasma, increasing plasma concentrations of saturated (SFAs), monounsaturated (MUFAs), and polyunsaturated FAs (PUFAs) (101). SFAs and MUFAs promote PDAC progression (102). However, a study performing transcriptomics and metabolomics suggested that lipase and a panel of FAs were significantly decreased in PDAC, and the presence of two SFAs (palmitate and stearate) inhibited tumor cell proliferation (103). Therefore, the roles played by FAs in PDAC appear complicated and remain unclear. PDAC patients present with distinct phenotypes associated with cachexia development, such as adipose tissue loss prior to skeletal muscle wasting or the loss of adipose tissue alone (104). A recent report indicated that soft tissue changes are initiated in PDAC before skeletal muscle loss (19), and the significant loss of visceral adipose tissue has been observed in PDAC-CC (18). In a retrospective cohort study, PDAC-CC was associated with the accumulation of oleic acid in plasma, resulting from malnutritional compensatory mechanisms triggered by the lack of oleic acid uptake into tissue (105). In a pre-cachexia model, increased FA oxidation occurs before muscle mass reduction, suggesting that FA may serve as a dominant energy source in PDAC-CC (18, 106). Adipose tissue lipolysis contributes to circulating FAs and subsequent FA uptake and lipid accumulation in the muscle and tumor tissue, leading to eventual metabolic derangement and muscle wasting after a period of metabolic adaptation. Several lipolytic enzymes are elevated, such as adipose triglyceride lipase (ATGL) and hormone-sensitive lipase (HSL), further suggesting the occurrence of enhanced lipolysis. Increased ATGL and HSL activity correlate with tumor growth and WAT loss in cachexia (107) (**Figure 3**). Tumor progression is also associated with the shift from WAT to BAT, known as adipose tissue browning. BAT is a metabolic hallmark mediated by uncoupling protein 1 (UCP-1). In KPC and *Kras^LSLG12D/+^Trp53^f/f^* mice, adipose tissue browning is associated with increased UCP-1 expression and occurs prior to the onset of fat wasting, consistent with clinical observations (108). A few studies have suggested that fat loss is an early and precipitating event prior to muscle loss in PDAC-CC, even in the absence of muscle wasting (18, 19, 21, 33, 104, 105, 107). Clinical studies suggested that fat loss may serve as a driving force for cachexia mortality, emphasizing the important roles of adipocytes in PDAC-CC and supporting the need to monitor adipose in patients with CC (20, 21).

Tumors hijack organ and tissue function, causing muscle and adipocyte wasting. Enhanced glycolysis in tumors and the upregulation of lipolysis and proteolysis in wasting tissue counterbalance the reductions in muscle and fat under cachectic conditions (**Figure 3**). Wasting muscle and adipocytes are among the convergent metabolic adaptations induced by tumors to satisfy their metabolic requirements. Patients with PDAC and PDAC-CC exhibit distinct and heterogeneous metabolic changes. Tumor, muscle, and adipocyte tissues all act as endocrine organs involved in the regulation of metabolic homeostasis, consistent with the EOLT hypothesis. In addition to metabolic alterations, bi-directional feedback occurs between tumor tissues and other organs, driven by the oncogenes and mediators (8, 12, 21, 32, 62).

### 3.2 Genetic instability–driven cachexic phenotypes and experimental models

**Figure 4:**
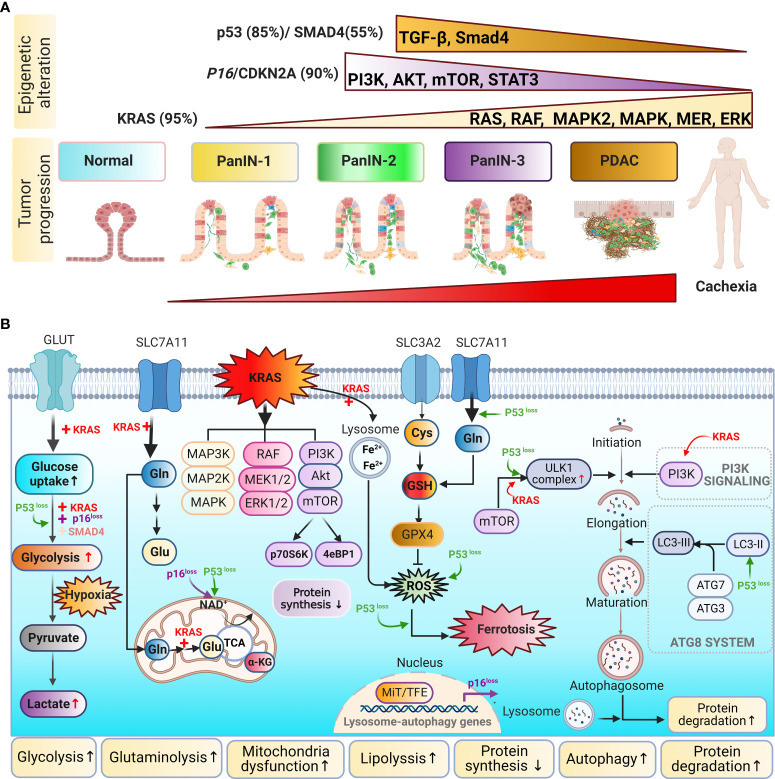
Metabolic remolding is influenced by genetic instability in PDAC. **(A)** Genetic mutations: PDAC is affected by high frequencies of aberrations and mutations in *KRAS*, *P16/CDKN2A*, *TP53*, and *SMAD4*. *KRAS* is involved in the RAF/mitogen-associated protein kinase pathway and the phosphoinositol 3-kinase (PI3K) pathway. *P16/CDKN2* mediates the PI3K/AKT/mTOR pathway. *TP53* influences the transforming growth factor-beta (TGF-β)/Smad4 pathway. **(B)** Mutated forms of *KRAS*, *TP53*, *P16/CDKN2*, and *SMAD4* promote glucose (Glc) uptake and enhance glycolytic flux, including the production of lactate (Lac). *KRAS* and *TP53* can both reprogram glutamine (Gln) metabolism to balance cellular redox homeostasis. Pancreatic cancer induces metabolic shifts, including increased glycolysis, lipogenesis, glutaminolysis, and autophagy, which are related to cachexia.

In PDAC, tumors become genetically unstable (**Figure 4**), and mutations in four oncogenes are common in PDAC: *KRAS* (>95%), *p16/CDNK2A* (> 90%), *TP53* (~70%), and *SMAD4* (55%) (8, 9, 62, 100). The hyperactivation of oncogenes (e.g., *KRAS*) and the downregulation of tumor suppressor genes (e.g., *TP53* and *CDKN2A*) promote tumor progression through the activation of various signal transduction pathways, including Wnt/Notch, c-Jun N-terminal kinase (JNK), PI3K, KRAS, and transforming growth factor (TGF)-β. A series of genetic and molecular events initiated by early oncogenic mutations in PanINs and later mutations in PDAC have been associated with metabolic alterations (109, 110). PDAC-CC is initiated by a metabolic shift in fuel utilization, in which glycolysis, proteolysis, and lipolysis increase and lipogenesis and protein synthesis decrease (**Figure 4**). During the pre-cachectic stage, patients experience these metabolic alterations as loss of appetite and impaired glucose metabolism before PDAC diagnosis or significant weight loss is apparent (**Figure 2**) (19, 67, 105). Early inflammatory signals may trigger the initial muscle and adipocyte wasting signaling cascades (9, 18, 21, 24).

*KRAS* is the most prevalently mutated oncogene, and *KRAS* mutations are considered to be dominant driver mutations in PDAC. Mutant *KRAS* regulates components of the mitogen-activated protein kinase (MAPK) and PI3K pathways to reprogram intracellular metabolism, including increasing glycolysis, by altering the levels of GLUT1 (111), HBP, and PPP (64, 112). Direct downstream effector cascades affected by *KRAS* mutations include the RAF–MAPK kinase (MEK)–extracellular signal–regulated kinase (ERK) and PI3K–AKT–mTOR pathways. The RAF–MEK–ERK pathway is considered among the most critical. The *KRAS^G12D^* mutation is frequently observed in pancreatic cells and promotes glycolysis *via* the upregulation of the MEK–ERK–HIF-1α axis. Elevated HIF-1α results in a feedforward loop between the insulin growth factor (IGF)-1 receptor, HIF-1α, and caveolin-1 to facilitate tumor progression and glycolysis (113).

Besides promoting high levels of glycolysis, *KRAS* upregulates glutaminolysis, allowing glutamine to be used as an additional carbon source for the TCA cycle (114). NEAAs, such as alanine, are alternative carbon sources that can support altered energy metabolism in PDAC (115). *KRAS* mutations increase glycolysis and the metabolism of amino acids, such as alanine and glutamine, activating downstream catabolic pathways, including proteolysis and lipolysis. Genetic mutations promote the recycling of wasting tissues to fuel cancer growth.

*KRAS* mutations also promote the generation of inflammatory cytokines, which shape the PDAC TME, including IL-6, IL-8, C–X–C motif ligand (CXCL)1, CXCL2, and CXCL5 (116, 117). Some cytokines/chemokines act in both autocrine and paracrine manners to support tumorigenesis and tumor angiogenesis (116).

*KRAS* activation leads to the loss of p16, accelerating NADH oxidation and supporting increased glycolysis through the production of NAD^+^ to support tumor growth (118). However, PDAC cells lack nutrient sensors, and mTOR complex 1 (mTORC1), a nutrient-sensing mechanism, is bypassed in PDAC. Bardeesy et al. proposed that autophagy is driven by the elevated expression of the microphthalmia transcription factor (MiTF) family members MiTF, TFE3, and TFEB in PDAC (119). The loss of *SMAD4* is another frequent event associated with PDAC progression, identified in approximately 50% of PDAC cases. *SMAD4* is a central component in the transforming growth factor (TGF-β) signaling cascade, and *SMAD4* loss enhances glycolysis by altering the expression of the glycolysis enzyme phosphoglycerate kinase (PGK) (120). The loss of *TP53* alters metabolism in PDAC by inhibiting mitochondrial respiration and simultaneously stimulating glycolysis. A recent study demonstrated that *TP53* rewires glucose and glutamine metabolism in malignant PDAC by preventing the nuclear translocation of glyceraldehyde 3-phosphate dehydrogenase (GAPDH) and stabilizing its function (121). Loss of *TP53* can reduce the expression of fructose-2,6-bisphosphate to promote the glycolysis cycle (122). Therefore, *KRAS*, *P16*, *P53*, and *SMAD4* have counterintuitive effects that promote tumorigenesis, further highlighting the complexity of interactions between genes and metabolisms in cancer progression and cachexia development.

These metabolic changes are consistently observed in numerous preclinical animal models of PDAC cachexia (**Table 4** **)**. Commonly used animal models of PDAC-CC include [1] intraperitoneal (IP) injections of PDAC cells, which localize to the pancreas; [2] orthotopic models of PDAC, in which cancer cells are injected directly into the pancreas; [3] patient-derived xenograft (PDX) models, in which a portion of a resected human pancreatic tumors are surgically attached to the mouse pancreas; and [4] genetically engineered mouse models (GEMMs) of PDAC. Up to 85% of PDAC patients suffer from CC, and approximately 30% of PDAC patients succumb to cachexia rather than tumor burden (137, 138). PDX and murine allograft models have been applied to study cachexia, resulting in the identification of Toll-like receptors 7/9 (TLR7/9), MyD88, and TGF-β as mediators of cachexia in PDAC (129, 137, 139, 140). Most PDAC-CC studies focus primarily on weight loss, muscle wasting, and the analysis of mRNA markers. A comparison analysis of subcutaneous, IP, and orthotopic PDAC animal models indicated that the implantation site is crucial when attempting to study PDAC-CC (137). Both IP and orthotopic implantation models develop more severe cachexia symptoms (such as muscle wasting, anorexia, and a decrease in locomotive activity) than the subcutaneous implantation model. The orthotopic animal model is histologically similar to PDAC patients, mimicking the TME associated with intact tumors, suggesting that the TME may be involved in cachexia induction. Studies in PDAC animal models have demonstrated that tumor-associated macrophages mediate muscle wasting *via* the activation of signal transducer and activator of transcription (STAT)3 signaling (134, 141).

**Table 4:** Genetically engineered mouse models (GEMMs) of pancreatic cancer-derived cachexia phenotypes.

	Orthotropic xenograft							Chemically induced	Genetically engineered	
**Model**	PANC-1	L3.6pl	S2-013 *(*SUIT-2*)*	COLO-357	MIA PaCa-2	Pan02	PDX	Gemcitabine-induced SW1990	KPC	KPP
**Method**	Injection: 1×10^6^ cells	Injection: 1×10^6^ cells	Injection: 5×10^5^ cells	1 mm^3^ sutured	5×10^6^ cells	(IP) 1×10^7^ cells	2 mm^3^ patient-derived	50 mg/kg, (IP) gemcitabine	KRAS^G12D^ P53^R172H^ PDX‐Cre^+/+^	KRAS^+/G12D^ Ptf1a^+/ER_Cre^ Pten^f/f^
**Mouse strain**	NSG mice NOD-SCID	NSG mice	Athymic nude mice	Athymic nude mice	Athymic nude mice	C57BL/6	NSG mice NOD-SCID	BALB*/c nu/nu* mice	C57BL/6	C57BL/6J Tamoxifen
**Age**	8-wk-old female	8-wk-old female	6–8-wk-old	6–8-wk-old	6-wk-old	6–8-wk-old male	8-wk-old female	4–6-wk-old	7–12-wk-old	4–5-wk-old
**Duration**	10 weeks	4–6 weeks	4 weeks	60 d	4 weeks	4*5* d	8–16 weeks	4 weeks	13–200 d	158 d
**Weight loss**	–	–	**+**	**+**	**+**	**+**	**+**	**+**	**+**	**+**
**Muscle wasting**	**+**	**+**	+*	**+**	**+**	+*	+*	–	+*/–	+*
**Muscle gene profiles**	FoxO1 Atrogin-1 MuRF1 SOCS3	FoxO1, Atrogin-1 MuRF1	MuRF1 Atrogin ZAG HSL	(+) INHBA	(+) SMAD2/3	MuRF1 Atrogin-1 ZAG myostatin	MuRF1 Atrogin-1 FoxO-1	N.A.	MuRF1 FoxO-1 pSTAT3 Atrogin-1	MuRF1 Atrogin-1 Atg5 Bnip3
**Note**	Chemokine **↑** IP10, MCP1, MIP2, RANTES and MIP1Β (spleen)		Metabolic alteration: ROS↑ Glutamine uptake↑	N.A.	Activin A **↑** via (+)PI3K/AKT (-)AKT/TORC	Metabolic alteration: proteolysis↑, lipolysis↑, via TGF-β/NF-κΒ	(+) JAK/STAT (+) FoxO (+)PI3K/AKT	(+) Anoxia Chemotherapy-induced mild cachexia	(+) Anoxia, Autophagy↑ *Orm1*↑, *Apcs*↑ (+) *Jak2/Stat3*	Clinical muscle wasting phenotype
**Inflammation evaluation**	TNFα**↑**, IL1β**↑**, IL6**↑**, and KC**↑** (murine IL8 homolog)		Not tested	TNFα**↑**	Not test	MCP-1, IL-6, TGF-β1	IL‐1β, IL-1α IL22, TNF, oncostatin M	Not tested	IL‐1β, IL-6 *Selp, Arg-1*	N.A.
**Metastasis**	–	–	N.A.	**+**	**+**	Not test	**+**	N.A.	−**/+**	**+**
**Ref**	(22, 123, 124)	(123)	(125, 126)	(127)	(128)	(129)	(130–132)	(133)	(21, 130, 134–136)	(130)

IP, intraperitoneal; N.A., not available; wk, week; d, day. *fat loss.

Most preclinical studies of CC use C-26 (colon cancer) and LLC (lung cancer) mouse models. However, these two models are associated with limitations (1): a limited interval between the onset of CC symptoms and animal death leaves only a small therapeutic window, and (2) translatability to humans may be limited, as the gene expression profiles in these mice did not correlate with those in human cancer tissue biopsies (130). However, GEMMs offer slower cachexia progression and early development than other cancer models, and PDAC-CC animal models are more translatable to humans than models using other cancer types. Therefore, animal models of PDAC-CC are clinically relevant. Preclinical PDAC murine models may be useful for understanding cachexia progression and evaluating therapeutic options for mitigating PDAC-CC. Establishing a model able to fully mimic the human condition remains necessary. Animal models can contribute to improving our understanding of the mechanisms driving tissue wasting for translation into new anti-cachexia therapies.

### 3.3 Pro-cachectic mediators and microRNAs

#### 3.3.1 Pro-cachectic mediators

**Figure 5:**
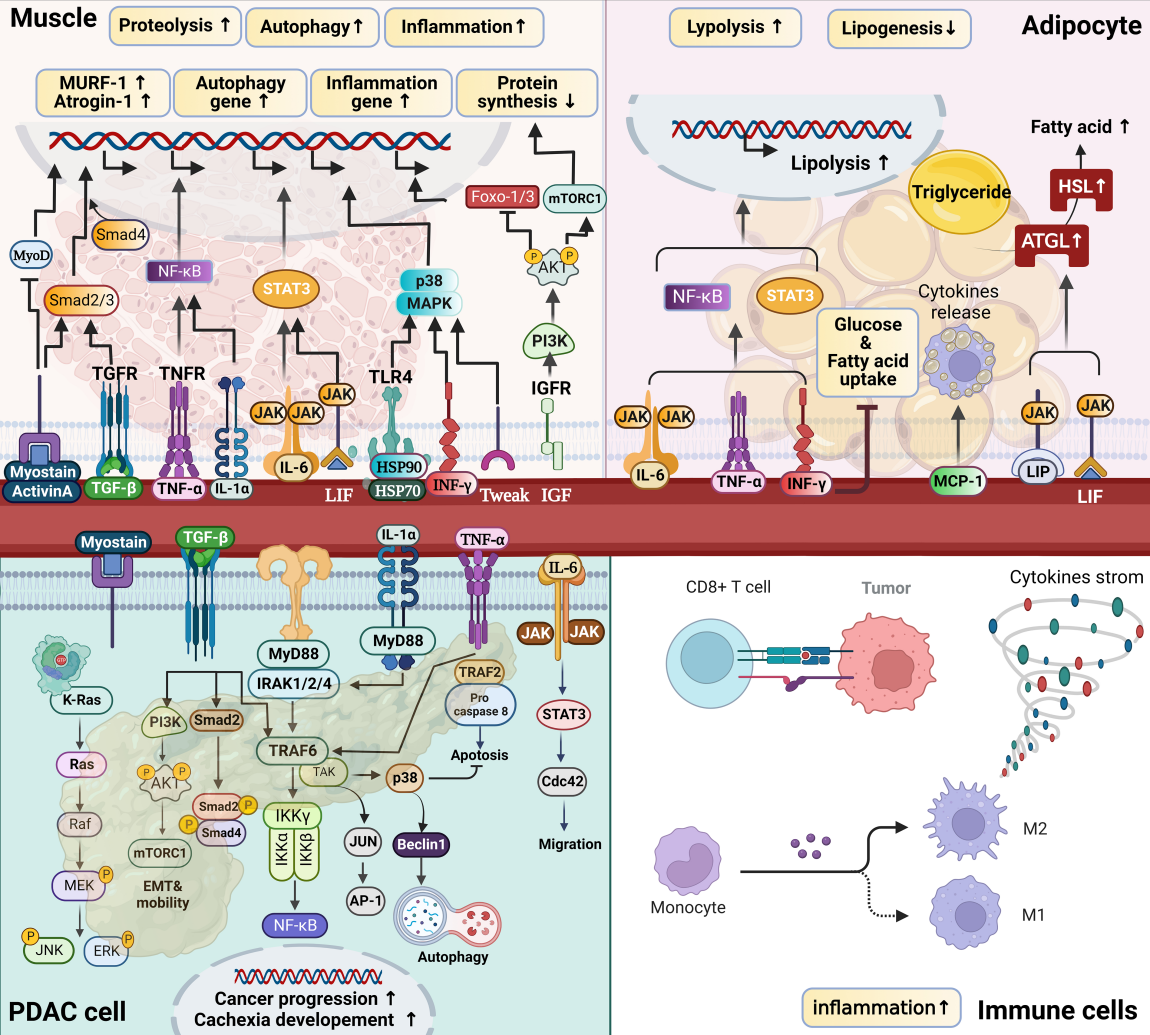
Pro-cachectic mediators of catabolism in PDAC-CC. Cachexia signals induce tissue catabolism by modulating gene expression profiles related to protein synthesis and degradation in muscle, lipid depletion, and tumor progression, primarily *via* the nuclear factor kappa B (NF-κB) and Janus kinase (JAK)–signal transducer and activator of transcription (STAT) pathways. In tumors, multiple receptors, including the Toll-like/IL1 receptors (TIRs), tumor necrosis factor (TNF), transforming growth factor-beta (TGF-β), and interleukin receptors (IL-6R being the best-studied), utilize overlapping and distinct signal transduction mechanisms to affect cellular outcomes, including increased cytokine production, proliferation, survival, migration, autophagy, and resistance to chemotherapy and immune surveillance. In addition to cytokines in tumors, circulating cytokines affect muscle and adipocytes, resulting in various metabolic alterations. For example, myostatin/activin A binds to type II receptors (ActRIIB), leading to Smad2/3 phosphorylation and the recruitment of Smad4, which results in muscle wasting. Simultaneously, myostatin/activin A signaling inhibits AKT activity and suppresses FoxOs phosphorylation, activating the ubiquitin–proteasome and autophagy–lysosome systems. IL6 binds to receptors to activate JAK/STAT3 signaling, increasing protein degradation. TNFα and IL1 signaling activates the IκB kinase (IKK)–NF-κB axis to initiate proteasome-mediated protein degradation. Higher levels of tumoral and stromal IL-1β expression result in a feedback circuit that attributes to cancer progression and cachexia development.

Endocrine organs and cells synthesize biologically active compounds that are released directly into the circulation and interact with other cells. Cachexia-associated inflammation is influenced by numerous bioactive molecules, such as TNFα, IL-1, IL-6, and IL-8 (**Table 5** and **Figure 5**). Cachexia-affected organs can act as autocrine or paracrine organs, releasing factors into the bloodstream to promote systemic crosstalk. These cytokines have multifactorial effects, triggering a hypercatabolic feedforward loop between the tumor, adipose tissue, and muscle mediated by the NF-κB and Janus kinase (JAK)/STAT pathways (29, 174, 175) (**Figure 5**). NF-κB and JAK/STAT activation enhance lipolysis, downregulate lipogenesis, and stimulate the catabolism of lean body mass (12).

**Table 5:** Pro-cachectic mediators in PDAC-derived cachexia.

Cachectic mediator	Source	Action	Model of the study	Function	Ref
**Pro-inflammatory**					
**IL-1 α**	Tumor	Autocrine	AsPC-1, PANC-1, Capan-1, CFPAC-1, MDAPanc-3, and MDAPanc-28	● IL-1α activates AP-1 and nuclear factor-ΚB (NF-κB) pathways driving carcinogenesis.	(142)
	Tumor Macrophage Spleen	Autocrine Paracrine	C57BL/6J-congenic KPC model, Orthotopic L3.6pl xenografts (NSG), Orthotopic PANC-1 xenografts	● IL-1α, a catabolic mediator, activates the STAT3 signaling pathway and contributes to myofiber atrophy.	(123, 134)
	Tumor	Paracrine	KPC and IL-1R1 knockout C57BL/6J	● Acting in a paracrine manner, activates NF-κB signaling and expression of LIF in iCAFs.	(143)
	Tumor	Paracrine	PANC-1 and MIA-PaCa2 Orthotopic patient-derived xenograft BALB/c bearing MiaPaCa-2 KCP model, PDAC specimens, n=100	● IL-1α induces inflammatory factors (IL-6 and CXCL8) that lead to JAK/STAT activation.	(144, 145)
**IL-1β**	Tumor, CAFs Serum	Autocrine Paracrine	Orthotopic PANC-1 xenografts, Capan-1 and PANC-1, Capan-1 xenograft, MIA-PaCa2/CAF xenograft, KCP model PDAC patients: 27 PDAC-CC, total=89	● Increased IL-1β levels are a poor prognosis marker. ● Activates IRAK4 and NF-κB, supports cancer progression and chemoresistance.	(146, 147)
**IL-6**	Tumor Spleen Serum	Paracrine	Orthotopic L3.6pl xenografts (NSG), Orthotopic PANC-1 xenografts, PANC-1 and T3M4 PDAC specimens (19 PDAC-CC, total=100) PDAC patients (85 PDAC-CC, total 126) PDAC patients (25 PDAC-CC, total 55)	● Increases IL-6 in tumor and spleen, associated with muscle wasting and systemic inflammation. ● IL-6 acts as a poor prognosis marker and a prominent cachexia-associated factor.	(68, 123, 148–151)
	Tumor	Paracrine	KPC and KPC IL6^KO^	● IL-6 causes adipocyte lipolysis and muscle steatosis, dysmetabolism, and wasting.	(21)
	Serum	Paracrine	PDAC serum, n=136 (a retrospectively studied)	● Higher IL-6 levels in tumor and serum mediate muscle wasting and cancer progression.	(152)
	Macrophages Fibroblasts Mast cells T cells	Paracrine	Review article	● Acute‐phase response (inflammation). ● Suppresses food intake.	(153)
**IL-8**	Tumor	Paracrine	PDAC sample n=8 (organoid culture)	● IL‐8 is associated with worse survival and muscle wasting.	(154)
	Serum Tumor	Paracrine	PDAC plasma (55 PDAC, total= 127) L3.6pl and COLO-357 PDX model C57BL/6J intraperitoneal injection IL-8	● Elevated serum IL-8 level significantly correlates with cachexia and sarcopenia. ● IL-8 is released from human PC cells in initiating atrophy of muscle cells *via* CXCR2-ERK1/2.	(81, 155)
**TNFα** **(Cachetin)** **(Cachexin)**	Tumor	Paracrine	CHO/TNF-20 cells implanted on nude mice.	● TNFα induces muscle wasting. ● TNFα inhibits both adipocyte and skeletal myocyte differentiation.	(70)
	Serum	Paracrine	PDAC patients (n=63)	● Increased TNFα levels in plasma correlated with poor nutritional status in advanced PDAC.	(156)
	Tumor, Spleen	Autocrine Paracrine	L3.6pl subcutaneous xenograft Orthotopic PANC-1 xenografts	● Elevated TNFα is associated with PDAC cachexia.	(123)
	Macrophage Lymphocyte	Paracrine	Review article	● Mediates muscle wasting, liver lipogenesis, insulin resistance, anorexia, and inflammation	(153)
**TGF-β**	Serum	Paracrine	KPC mice bearing Panc02 KPC mice bearing FC1242 tumor (Intra-cardiac injection)	● TGF-β is a potent inducer of muscle atrophy, weight loss, and fat loss (increasing catabolism: proteolysis and lipolysis).	(129)
	Tumor	Paracrine	AsPC-1, MIA-PaCa2, BxPC-3, PANC-1, and CFPAC-1 Orthotopic xenograft mouse bearing AsPC	● TGF-β downregulation suppresses tumor growth and muscle wasting.	(157)
**INF-γ**	Serum	Paracrine	PDAC serum samples, n=90	● Increased serum INF-γ is associated with cachexia.	(158, 159)
	Lymphocytes Natural killer	Paracrine	Review article	● Catabolic effects	(153)
**MyD88**	Serum	Paracrine	KPC mice	● MyD88 trigger inflammation that influences cachexia development.	(140)
**Other factors**					
**ZIP4**	Tumor	Autocrine Paracrine	Orthotopic nude mice bearing AsPC-1 Orthotopic nude mice bearing BxPC-3	● ZIP4 promotes PDAC progression and muscle wasting by activating CREB- RAB27B.	(148)
	Tumor	Paracrine	Orthotopic xenograft mouse bearing AsPC (stable cell line: AsPC-shZIP4-Pre373)	● ZIP4 causes muscle wasting *via* PHLPP2-AKT-TGFβ Signaling Axis.	(157)
**ZIP14**	Muscle	Paracrine	C57BL/6 mice bearing Panc02 C57BL/6 mice bearing FC1242 tumor	● High levels of ZIP14 in muscles correlated with muscle wasting in PDAC.	(160)
**Activin/** **Activin A**	Serum	Paracrine	PDAC specimens, N=34 KPC model	● Activin A mediated triglyceride degradation and accelerated visceral adipose wasting.	(18)
	Stroma Tumor	Paracrine	PDAC tissue microarrays n=63 Tumor samples *vs*. adjacent-normal KPC mouse model	● High activin A expression in stroma correlated to a worse prognosis.	(161)
	Tumor	Paracrine	KPC model	● PDAC tumors choreograph a systemic activin A response that correlated with muscle wasting.	(162)
	Tumor Serum	Paracrine	PDAC specimens, N=124 GEMM KPC mouse model	● Activin with hormone regulation shows a preferential driver of muscle wasting in males.	(163)
**ZAG**	Adipocyte	Paracrine	Review article	● Muscle wasting, insulin resistance, inflammation ● Anorexia	(153)
**UCP-1**	BAT, WAT	Paracrine	PDAC samples, N=8	● Uncouples oxidation of mitochondrial fatty acids. ● Thermogenesis and WAT browning.	(18, 164, 165)
**LMF**	Tumor	Paracrine	MAC16-murine model	● Lipid mobilizing factor (LMF) secreted from the tumor acts directly on adipose tissue with the release of FFA and glycerol.	(166)
**Caveolin-1**	Tumor	Paracrine	Athymic mice bearing MIA-PaCa2	● Cav-1 in PDAC stimulated IGF1R/IR, and glycolysis triggered cachectic states.	(167)
**MCP-1**	Serum	Paracrine	PANC-1 cells PDAC patients (n=70)	● MCP-1 led to inflammation and induced lipolysis by activating hormone-sensitive lipase (HSL). ● Suggesting MCP-1 acts as a biomarker of cancer cachexia.	(75, 168)
**DAMPs**	Serum tumor	Paracrine		● Extracellular HSP70 and HSP90 function as DAMPs and provoke an innate immune response through activation of TLR7/9 and TLRs. ● Muscle wasting.	(162, 169)
**PAMPS**	gut microbiota	Paracrine		● Inflammation and muscle wasting.	(170, 171)
**Hormone**					
**Glucocorticoids**	Serum	Paracrine	KCP murine model	● Hight GC content mediated skeletal muscle catabolism and hepatic metabolism during cancer cachexia.	(172)
**PTH**	Tumor	Paracrine	Review article	● Hypercalcemia ● Pro-cachectic factor, Pro-inflammatory stimulant	(153)
**PTHrP**	Tumor	Paracrine			
	Serum	Paracrine	624 patients	● Regulating UCP1 expression reverse muscle and adipose tissue loss	(173)
**Leptin**	Adipocyte	Paracrine	Review article	● Leptin reduces appetite and increases energy expenditure.	(88)

TNF-α, tumor necrosis factor-alpha; TGF-β, transforming growth factor-beta; IFN‐γ, interferon‐γ; IL, interleukin; PTH, parathyroid; PTHrP, parathyroid hormone‐related protein; UCP‐1, uncoupling protein‐1; WAT, white adipose tissue; ZAG, zinc‐a2 glycoprotein; DAMPs, danger-associated molecular patterns; PAMPS, pathogen-associated molecular patterns; LMF, lipase maturation factor; MCP-1, monocyte chemoattractant protein-1; ZIP, zinc-regulated, Iron-regulated transporter-like protein; IL, interleukin.

A salient feature that distinguishes PDAC from other *KRAS*-mutant cancers is an extensive fibro-inflammatory stroma, which accounts for 80%–85% of the tumor bulk. These stromal cells are recruited and reprogrammed by PDAC cells during cancer progression and cachexia development. Secreted factors (**Table 5**) enable these cells to communicate with PDAC, creating a dynamic feedback circuit associated with intrinsic *KRAS* signaling in PDAC cells (**Figure 5**).

Various circulating pro-inflammatory cytokines have been implicated in PDAC-CC, including IL-6, IL-1, IL-8, TGF-β, and TNFα …etc (detailed in **Table 5**) (12, 21, 29, 123). These cytokines likely derive from various sources and result in systemic effects (123, 176). Oncogenic *RAS* drives the expression of multiple inflammatory cytokines, including IL-1 and IL-6 (21, 146). Inflammatory cytokines released by tumor cells may be prominent cachexia-associated factors that regulate autocrine and paracrine function, promoting tumor progression and cachexia development. For example, IL-6 plays autocrine roles in supporting tumorigenesis *in vivo* and induces weight loss and inflammation in cachexia *via* a paracrine manner (21, 68, 152). Other cytokines, such as IL-1, IL-8, TNFα, and INF-γ, have also been associated with weight loss and poor survival in PDAC (148, 177, 178). TNF-α and IL-1 can induce anorexia, producing both hypercatabolic and anorexigenic effects (**Figures 1** and **5**). Circulating IL-1β promotes NF-κB activation in the hypothalamus, enhancing glucocorticoid production and resulting in catabolic effects in both muscle and adipose tissue. TGF-β1 can induce proteolysis through the E3 ligase atrogin-1 in animal models. TGF-β inhibition improved muscle wasting in the KPC model (129). Activin A and myostatin belong to the TGF-β superfamily and are associated with muscle wasting through the activation of the Smad2/3 pathway, which decreases AKT–mTOR-mediated protein synthesis and enhances ubiquitin ligase–mediated proteolysis (18, 128). These cytokines drive diverse catabolic processes across multiple cells and organs, forming a catabolic feedforward loop (**Figure 1** **)**.

In muscle tissue, the JAK–STAT and NF-κB pathways are the dominant catabolic pathways activated by circulating IL-6 and TNFα in muscle wasting (174). IL-6 induces NF-κB activation, which can also upregulate ubiquitin-mediated proteasomal degradation in wasting (179). Proteolysis is a prerequisite for muscle wasting, and both lipolysis and adipopenia may occur prior to muscle loss. Lipolysis results in increased circulating FFAs, triggering the secretion of Atrogin-1 and MuRF1, which induce muscle atrophy.

In adipocytes, lipolysis plays a substantial role in increasing the catabolism of stored fat. ATGL and HSL act to reduce fat to its component FAs, leading to the loss of body mass. Secreted IL-6 can trigger browning by inducing UCP-1 expression in adipocytes (18, 180). The catabolic effects of IL-6 on WAT *in vitro* are mediated through the JAK/STAT3 and NF-κB pathways (21, 181).

#### 3.3.2 Epigenetics modulation (miRNAs)

**Figure 6:**
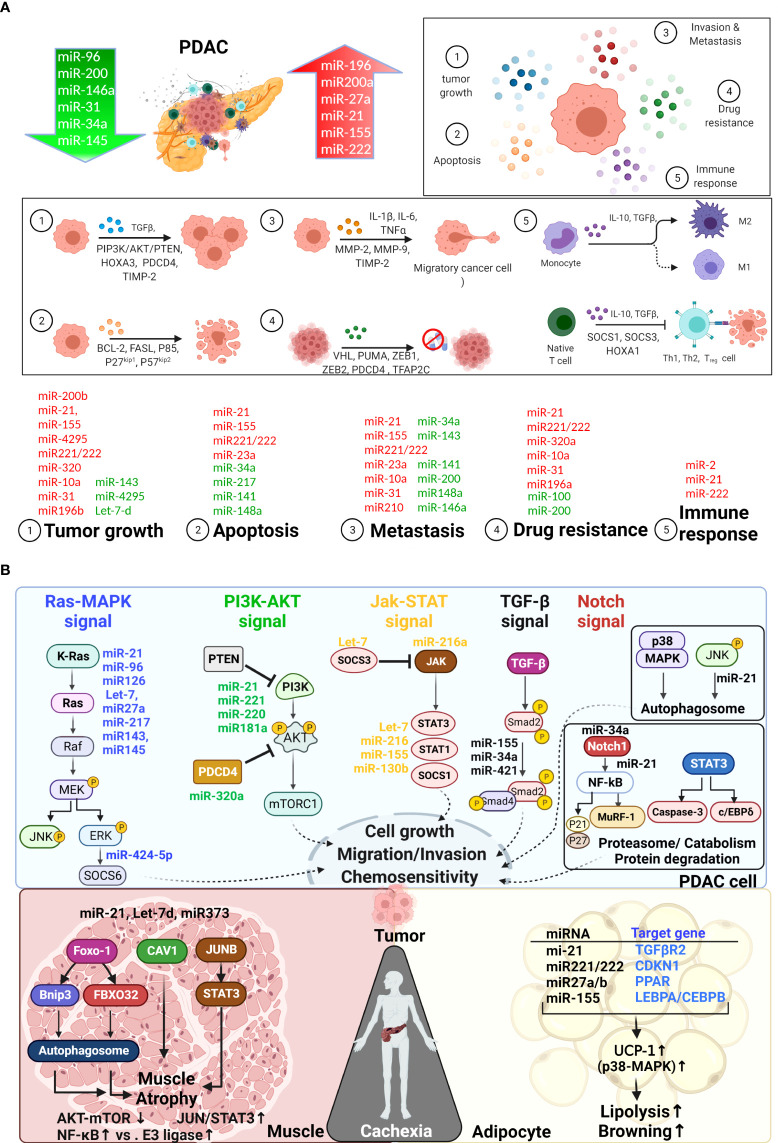
Mechanistic role of miRNAs in PDAC cachexia. **(A)** Downregulated microRNAs (miRNAs) are indicated in green, and upregulated miRNAs are indicated in red. Based on the literature, miRNAs participate in the regulation of PDAC progression and metastasis, overcoming host immune responses, and the development of chemoresistance. **(B)**. miRNAs associated with PDAC-CC are primarily involved in KRAS-MAPK, PI3K-AKT, JAK-STAT, and TGF-β, NF-κB and p38-MAPK signaling pathway … etc. miRNAs can be detected in tumor and serum and mediate crosstalk in the tumor microenvironment between tumor, muscle, and adipocytes, which are associated with the development of PDAC-CC. Some microRNAs are tissue specific. For example, miR-21 (TLR7-JUN), miR-155, let-7d and miR373 are specific contributed to muscle wasting in PDAC-CC (see **Table 6** in detail). The most common genes, such as IL-6R, FOXO1, PDK4, and ZIP14, had been associated with muscle wasting in cachexia. In adipocytes, specific microRNAs may mediate the transcription factors C/EBPβ/δ, C/EBPα, and PPARγ, resulting in adipogenesis.

Genetic instability and epigenetic changes are both involved in pancreatic oncogenesis and cachexia development (**Table 6**). Recently, miRNAs, small non-coding RNAs 19–25 nucleotides in length, have been identified in an increasing number of biological processes, including *KRAS* signaling and the JAK–STAT, PI3K–AKT, notch, and TGF-β signaling pathways (**Figure 6A**). These influences contribute to the control of several cancer-related processes in PDAC, such as tumor growth, apoptosis, metastasis, drug resistance, and the immune response. In addition to roles in oncogenesis and tumorigenesis, aberrant miRNA expression may affect cytokine production or directly alter metabolic processes, resulting in a metabolism remodeling that facilitates PDAC progression and cachexia development (see **Figure 6** and **Table 6**).

**Table 6:** MicroRNA (miR) expression levels and functions in pancreatic ductal adenocarcinoma (PDAC) and PDAC cachexia.

miRNA	Pathway	Target genes	Type of study	Location	Biological significance	Ref
miRNA-21 ↑	PI3K–AKT KRAS EGFR Cell cycle Apoptosis TGF-β	G12D, p27, p57, FOXO1, Bcl-2, FasL, PI3K, AKT, PTEN, RECK, SPRY2, P85, VHL, PDCD4, c-Jun	*In vitro*: PC1, Panc-2, and MIA-PaCa2, PANC-1, HS766T, HPAF-II, BxPC-3, Mpanc-96, PL45, Panc03.27, Panc10.05 *In vivo*: PDAC *vs*. healthy pancreatic duct tissue	In MV In tumor In blood	Promotes cell growth, invasion, migration and chemoresistance. Upregulation of miR-21 may inhibit myogenesis *via* regulation of IL6R, PTEN, and FOXO3 signaling. miRNA-21 promote muscle proteolysis *via* TLR7-JUN pathway.	(182–197)
miR-155↑	JAK–STAT TP53 MAPK-p38	TP53INP1, SOCS1, SOCS3, FOXO3a, TP53-induced nuclear protein 1 gene, RHOA, SMAD1/5, ZNF652	*In vitro*: BxPC-3, PANC-1, Capan-2, HS766T, HPAF-II, BxPC-3, Mpanc-96, PL45, Panc03.27, Panc10.05 *In vivo*: nude mice bearing MIA-PaCa2 PDAC *vs*. healthy pancreatic duct tissue	In MV In tumor In blood	Promotes tumor progression, invasion, and migration and mediates apoptosis. Higher miR-155 contributes to cachexia through the inhibition of negative feedback loops of SOCS1. miR-155 mediates TNF-Α showing a pro-inflammatory effect.	(182, 190, 198–204)
miR-221/222 ↑ (Tumor)	PI3K–AKT TP53 P16/P27 Cell cycle	MMP-2, MMP-9, TIMP-2, PTEN, P27kip1, P57kip2, PUMA, Cdk4, p16, E2F, CDKN1B, MDM2, ICAM-1, BIM, SOD2, STAT5A	*In vitro*: BxPC-3, SW-1990, PANC-1, MIA-PaCa2, HS766T *In vivo*: PDAC *vs*. healthy pancreatic duct tissue Advanced pancreatic cancer with lymph node metastasis	In tumor	Promotes tumor progression, proliferation, and invasion. Inhibits apoptosis and induces chemoresistance. In C2C12 cell models, downregulated miR221/222 is observed which is associated with cachectic and sarcopenic condition vis MyoD-myomiRs regulatory pathway.	(188, 202, 205–208) (209)
Let-7 ↓	JAK–STAT KRAS	STAT3, SOCS3, N-cadherin, ZEB1	(PDAC) Biopsy specimens	In tumor In serum	Tumor growth and migration. Lower serum levels of let-7d correlated with poor overall survival in PDAC.	(210) (211)
Let-7d↑	KRAS mTOR	KRAS PGR, RPS6KA6, SFRP4	*In vivo*: Pancreatic tissues (PDAC) Biopsy specimens Skeletal muscle biopsies	In tumor In muscle	Cell proliferation, migration, invasion, and apoptosis. Upregulation of let-7d affects muscle cell proliferation and myogenic differentiation which leads to skeletal muscle wasting.	(212–214) (215)
circANAPC7/miR-373	PHLPP2–AKT–TGF-β	ZIP4 promoter	*In vivo*: Orthotopic xenograft mouse bearing MIA-ZIP4-EV/circANAPC7	N.A.	Suppresses tumor growth and muscle wasting in PDAC.	(157)

MV, microvesicles; EMT, epithelial–mesenchymal transition; N.A., not available.

Recent studies have detected miRNAs in serum, plasma, tissue, and tumors (**Table 6**). Studies indicates that miRNAs are commonly found in various EVs, such as exosomes, apoptotic bodies, microvesicles (MV), and lipoproteins, allowing them to target cells and contribute to intercellular signaling through endocrine, paracrine, and autocrine pathways (182, 183, 198, 199, 215–217). Losses in muscle proteins and fat mass are the most important signatures of cachexia and can result in the generation of microvesicles containing miRNAs (see **Figure 6B**: bottom panel). Most studies have identified miRNAs expressed in tumor cells; however, some miRNAs are tissue‐specific or tissue‐enriched, involved in either the active or passive stimulation of metabolic changes and inflammatory responses (**Table 6**). In PDAC-CC, miR-21, miR221/222, miR27a and miR155 are commonly correlated with muscle and adipocyte wasting through the transcription E3 ubiquitin ligases (mediated by FoxO3 or FoxO1 in muscle), JUN–STAT3, or TGF-β; (**Figure 6B**: bottom panel). Given the roles of miRNAs in gene expression and the regulation of inflammatory responses and metabolic reprogramming, additional study of miRNAs remains necessary. An ongoing observational trial (NCT05275075) aims to analyze the miRNA profiles in patients with PDAC-CC. The causal roles of miRNAs and molecular mechanisms in cachexia remain under debate and require further discussion and study; however, therapeutic approaches for modifying multiple targets have been suggested (218, 219). The study of miRNAs could also contribute to the development of diagnostic or prognostic biomarkers and new targets for cachexia prevention or treatment.

## 4 Conclusion

The high prevalence of PDAC-CC may be associated with the unique genetic background (*KRAS* mutations) and modulators in PDAC, which mainly exacerbate metabolic disruptions, leading to cachexia development. Systemic metabolic alterations mediated by pro-cachectic factors, systemic inflammation, and epigenetic changes, highlighting that PDAC is a systemic disease rather than a single-organ defect. PDAC can induce metabolic disruptions in organs beyond the pancreas. We applied the EOLT hypothesis (33) to emphasize the systemic effects of PDAC, leading to tissue wasting in PDAC-CC. Currently, no FDA-approved agent is able to treat cachexia, although potential treatments are listed in **Table 7**. However, further studies remain necessary to generate foundational knowledge for the development of additional therapies or understanding the molecular mechanisms of PDAC-CC.

**Table 7:** Therapy for PDAC cachexia.

Target route	Drug	Target/mechanism	Cachexia/PDAC-CC	Biological significance	Status	Ref
Cachexia mediator/ pathway	NSAID agents thalidomide	Cytokine	Cachexia	Altered cytokine production.	Experimental Therapy	(220)
				Stabilized lean body mass.		(221)
	Infliximab etanercept	TNFα inhibition/ Mediating MuRF1 and Atrogin-1 expression in muscle	Cachexia PDAC-CC	No significant improvements in cachectic patients.	Phase II	(221–225)
				TNFΑ blockade failed to improve muscle wasting.	Phase I/II	(225, 226)
	Landogrozumab LY2495655 monoclonal antibodies (MoAbs)	Myostatin antibody/ Alk4/5/7/Smad and PI3K/AKT/mTOR pathways	PDAC-CC	Increased lean body mass. No benefits on overall survival. Myostatin antibody (LY2495655) with standard-of-care chemotherapy failed to confer additional clinical benefits (overall survival).	Phase II	(227, 228)
					NCT03207724	
	Tocilizumab	anti-IL-6R mAb/ JAK/STAT3 pathway	PDAC-CC	Improved appetite and body weight	Experimental	(229, 230)
	Clazakizumab ALD518 BMS-945429		PDAC-CC	Improved anorexia. Failed to reverse muscle atrophy.	Phase I/II	(231)
			Cachexia	Improved lean muscle mass, lung symptoms, and fatigue score.		
	AG490/ Ruxolitinib	JAK/STAT3/ Reduce proteolysis in muscle cells	PDAC-CC	Alleviated cancer cachexia and skeletal muscle wasting.	Phase II/III NCT00952289 NCT01423604	(232)
				Ruxolitinib plus capecitabine was well tolerated, but no improvement in survival.		(233)
	Trabederson AP 12009	TGF-β2 antagonist	Cachexia	Tumor suppression.	Phase II	(139, 234, 235)
				Effect on anorexia.	Experimental	(236)
	Bimagrumab (BYM338)	Anti-ACVR2 antibody	PDAC-CC	Increased lean body mass. Improved in thigh muscle volume (TMV), inter-muscular adipose tissue (IMAT) and subcutaneous adipose tissue (SCAT)	Phase II NCT01433263	(139, 237, 238)
	Anamorelin ONO-7643 ANAM	Ghrelin receptor agonist	PDAC-CC	Improved food intake, appetite, adiposity, and lean body mass. Adverse events (hyperglycemia, nausea, and dizziness) exist.	Phase III NCT01395914 NCT04844970 NCT03035409 NCT03637816 NCT01387269 NCT01387282	(239–243)
	Omeprazole	Hsp70/90	Cachexia	Prevented loss of muscle function.	Experimental	(244)
	MicrSoy-20	Gut Microbiota	PDAC-CC	Improved fatigue and appetite loss.	NCT04600154	(245)
	Espindolol MT-102	5-HT1aR/β2 agonist	Cachexia	Improved weight loss and fat-free mass. Acts as a pro-anabolic, anti-catabolic, and appetite-stimulator.	Phase II NCT01238107	(246)
	IMO-8503	TLR7/8/9 antagonist	Cachexia	Suggested a potential therapy for cancer cachexia.	Animal model	(247)
	R848	TLR7/8 antagonist	PDAC PDAC-CC	R848 induces anti-tumor responses and attenuates cachexia, improving the survival.	KPC model	(248)
Hormonal	RU38486	Glucocorticoid antagonist	Cachexia	U38486 was ineffective in muscle wasting.	Tumor-induced animal model	(249, 250)
Nutritional interventions	Ketogenic diet	Metabolism	PDAC-CC	Reversed metabolic alterations and reduced glycolytic flux and glutamine catabolism.	Experimental	(132)
	Glutamine Arginine β-hydroxy-β-methylbutyrate	Metabolism	Cachexia	Shifted away from proteolysis and increased fat-free mass.	Experimental	(251)
				Failed to improve lean body mass.	Phase III	(252)
	BCAA	Metabolism	Cachexia	Stimulated muscle protein synthesis Inhibited proteolysis	Clinical trial NCT03253029	(253–255)
	BCAA β-hydroxy-β-methylbutyrate	Metabolism	Cachexia	Fat mass content increased with no change in fat-free mass.	NCT03285217	(256, 257)
	n-3 polyunsaturated fatty acids		PDAC-CC	Stabilized weight and appetite in pancreatic cancer patients.	NCT03751384	(258–264)
			Cachexia	Resist muscle wasting and improve the survival.	N.A.	(265)
PERT	Pancreatic enzymes	EPI	PDAC-CC	Weight gain, limited weight loss.	NCT02127021	(266, 267)

EPI, exocrine pancreatic insufficiency; CC, cancer-derived cachexia; TNFα, tumor necrosis factor-alpha; BCAA, branched-chain amino acids; TLR, Toll-like receptor; 5-HT, 5-hydroxytryptamine; TGF, transforming growth factor, NSAID, non-steroidal anti-inflammatory drugs; Pancreatic enzyme replacement therapy, PERT.

## Author contributions

Y-CY and AA drafted manuscript. L-MC, W-CCe, J-CY, H-CL and W-CCa participated in discussion, literature review, supported, and edited manuscript and W-LM initiated and supported the concept. Y-SS and W-LM edited, and final approved the manuscript. All authors contributed to the article and approved the submitted version.

## Funding

This work was supported by Ministry of Sciences and Technology, Taiwan (MOST 107-2314-B-039-011; MOST 108-2320-B-039-017; MOST 108-2314-B-039-043-MY3; MOST 108-2314-B-039-052, MOST 111-2320-B-039-011, MOST 111-2314-B-039-062-MY3, MOST 110-2314-B-039-046), and National Health Research Institute, Taiwan (NHRI-EX109-10705BI, NHRI-EX111-11110BI), and China Medical University/Hospital (DMR-109-019, CMU109-MF-26, CMU111-MF-91, CMU111-MF-41, DMR-111-118, DMR-111-238, DMR-110-025, and DMR-111-204) for Dr. Wen Lung Ma, and MOST 110-2314-B-006-106 and MOST 111-2811-B-006-017 for Yan Shen Shan.

## Acknowledgments

Authors of this manuscript comply with the ethical guidelines for authorship. All figures created with BioRender.com.

## Conflict of interest

The authors declare that the research was conducted in the absence of any commercial or financial relationships that could be construed as a potential conflict of interest.

## Publisher’s note

All claims expressed in this article are solely those of the authors and do not necessarily represent those of their affiliated organizations, or those of the publisher, the editors and the reviewers. Any product that may be evaluated in this article, or claim that may be made by its manufacturer, is not guaranteed or endorsed by the publisher.

## Glossary

**Table:**

PDAC	Pancreatic ductal adenocarcinoma
PDAC-CC	PDAC-derived cachexia
EOLT	endocrine organ–like tumor
QoL	quality of life
OS	overall survival
CC	cachexia
IL	interleukin
TNF-Α	tumor necrosis factor-alpha
BMI	Body Mass Index
CRP	C-reactive protein
SMI	Skeletal Muscle Index
BIA	bioelectrical impedance analysis
CXI	cachexia index
TME	tumor microenvironment
PI	pancreatic insufficiency
OXPHOS	oxidative phosphorylation
PanIN	pancreatic intraepithelial neoplasia
PPP	pentose phosphate pathway
TCA	tricarboxylic acid
HBP	hexosamine biosynthesis pathway
HK1/2	hexokinase ½
LDHA	lactate dehydrogenase A
LDH	lactate dehydrogenase
GLUT1	glucose transporters 1
MCT	monocarboxylate transporters
GFPT	fructose-6-phosphate amidotransferase
HIF-1	hypoxia-inducible factor-1
FA	fatty acid
WAT	white adipocyte tissue
BAT	bBrown adipose tissue
ROS	reactive oxygen species
NEAA	nonessential amino acid
AA	amino acid
BCAA	branched-chain amino acid
Q/E	glutamine/glutamate
GLS1	Glutaminase 1
GDH	glutamate dehydrogenase
Α-KG	Α-ketoglutarate
GOT1	aspartate transaminase 1
NF-Κb	nuclear factor kappa B
mTOR	mammalian target of rapamycin
MuRF-1	muscle RING-finger protein-1
MAFbx	muscle atrophy F-box
CoA	coenzyme A
FASN	fatty acid synthase
HMGCR	3-hydroxy-3-methylglutaryl coenzyme A reductase
SFAs	Saturated fatty acids
MUFAs	monounsaturated fatty acids
PUFAs	polyunsaturated fatty acids
ATGL	adipose triglyceride lipase
HSL	hormone-sensitive lipase
UCP-1	uncoupling protein 1
MAPK	mitogen-activated protein kinase
IGF	insulin growth factor
CXCL	C–X–C motif ligand
TGF-Β	transforming growth factor
GEMMs	genetically engineered mouse models
STAT3	signal transducer and activator of transcription 3
JAK	Janus kinase
INF-γ	interferon gamma
MV	microvesicles
EV	extracellular vesicles
FoxO	Forkhead box O
PIF	proteolysis-inducing factor
ZAG	zinc alpha 2-glycoprotein
LIF	leukemia inhibitory factor
TWEAK	TNF-related weak inducer of apoptosis
